# Potential Use of Grape Pomace for the Development of New Products: Study of Inorganic Elements Through Simulated Gastrointestinal Digestion

**DOI:** 10.3390/foods15122060

**Published:** 2026-06-07

**Authors:** Ana Paula Rebellato, Augusto César Costa-Santos, Raquel Fernanda Milani, Maiara Monteiro Azevedo, Juliana Azevedo Lima Pallone, Marcelo Antônio Morgano

**Affiliations:** 1 Institute of Food Technology, Av. Brasil 2880, Campinas 13070-178, SP, Brazil; raquel.milani@ital.sp.gov.br (R.F.M.); maiara.azevedo@hotmail.com (M.M.A.); morgano@ital.sp.gov.br (M.A.M.); 2 Department of Food Science and Nutrition, School of Food, Engineering, University of Campinas, Monteiro Lobato Street, 80, Campinas 13083-862, SP, Brazil; a265452@dac.unicamp.br (A.C.C.-S.); jpallone@unicamp.br (J.A.L.P.)

**Keywords:** bioaccessibility, phenolic compounds, agro-industrial by-product, estimated exposure, minerals

## Abstract

This study characterized eleven grape pomace varieties from São Paulo and Rio Grande do Sul (Brazil) in terms of proximate composition, total phenolic content, and the total and bioaccessible fractions of inorganic elements and phenolic compounds. On a dry basis, ash, protein, lipid, and carbohydrate contents ranged from 1.4–2.9%, 6.4–13.5%, 8.6–14.7%, and 72.1–83.6%, respectively. Total phenolic content ranged from 567 to 1843 mg GAE/100 g. The contents of essential elements (Ca, Fe, Zn, Cu, Mg, Mn, P, Na, and K) and trace elements (Al, Co, As, Se, Mo, Cd, Sb, Ba, Hg, and Pb) showed wide variation among the varieties studied. Regarding bioaccessibility, low values were observed for Fe, Zn, and Ca, while significant percentages were detected for Mg (27–30%), Mn (1–14%), P (16–26%), Cu (5.5–13%), Co (29–72%), Al (2–13%), and Ba (ND-9%), as well as levels of bioaccessible phenolic compounds (7–19%). The grape pomaces evaluated in this study presented variable levels of total phenolic compounds and minerals, although the bioaccessible fractions varied among the analyzed elements and compounds. These findings contribute to the characterization of Brazilian grape pomaces and indicate their potential applicability as value-added agro-industrial by-products in food formulations, while highlighting the importance of considering bioaccessibility when assessing their nutritional relevance.

## 1. Introduction

The Brazilian vitivinicultural sector is highly diversified. In addition to fine grapes (*Vitis vinifera*), which are widely used worldwide for both fresh consumption and processing, Brazil also employs American grape varieties (*V. labrusca*) and other species [1]. This diversity gives rise to production chains composed of fine, American, and hybrid grapes destined for table consumption, such as Niagara Rosada, Isabel, and Niagara Branca; grapes used for the production of fine wines; American and hybrid grapes employed in the manufacture of table wines, including *Moscato*, *Niagara Rosada*, *Bordô*, *Concord*, and *Isabel*; as well as grapes used for juice production, such as *Isabel*, *Concord*, and *Bordô* [1]. In addition, red grape varieties such as *Cabernet Sauvignon*, *Cabernet Franc*, *Merlot*, *Petit Sirah*, *Gamay Beaujolais*, and *Pinot Noir*, as well as white varieties including *Malvasia*, *Trebbiano*, *Sylvaner*, *Riesling*, and *Chardonnay*, are widely used in wine production and may exhibit distinct chemical and mineral compositions [2,3,4,5].

During grape processing, large amounts of pomace are generated. According to the literature, approximately 20% of the total grape weight is considered waste [6,7,8], and only a fraction of this material is reused, mainly for composting or animal feed. In general, processing companies do not benefit from this by-product; on the contrary, they incur operational costs associated with its disposal. As a result, large quantities of grape pomace are discarded, raising significant environmental concern [6,8,9].

Agro-industrial grape pomace comprises solid by-products such as stems, skins, seeds, and residual pulp, as well as filtered liquid fractions [7,10,11]. This residue presents a complex composition, consisting of water, proteins, lipids, carbohydrates, and fibers, in addition to compounds with relevant chemical and biological properties, including vitamins, phenolic compounds, and inorganic elements essential to human metabolism. Among these elements are calcium, iron, zinc, potassium, manganese, magnesium, copper, and phosphorus, which perform different physiological functions in the human body [3,4,7,12,13,14]. However, grape pomace may also contain potentially toxic trace elements, such as aluminum, chromium, cobalt, nickel, arsenic, cadmium, mercury, and lead, among others. Even at low levels of exposure, these elements may pose risks to human health. After absorption, such metals can be systemically distributed and affect different organs, with the main concerns being their lack of known biological function and their high potential for bioaccumulation [15,16,17,18,19,20].

Grape pomace also contains high levels of dietary fiber and polyphenols. Some authors have reported that approximately 70% of these compounds remain retained in this by-product during grape processing [21,22], making it particularly interesting from a nutritional standpoint [23,24]. The largest fraction of extractable phenolic compounds in grape pomace originates from the seeds, which are rich in flavan-3-ols, proanthocyanidins, and phenolic acids, whereas the skins predominantly contain flavonols and anthocyanins [25]. In plant matrices, phenolic compounds may also associate with carbohydrates, lipids, organic acids, and cell wall components. Therefore, the consumption of foods rich in phenolic compounds has been associated with several beneficial physiological effects, including anti-inflammatory, antiatherogenic, anticarcinogenic, antiallergic, antiviral, and antibacterial properties. These effects are mainly attributed to the antioxidant capacity of phenolic compounds and their ability to scavenge free radicals [3,7,23,26,27,28]. However, the biological properties and health benefits of phenolic compounds are directly related not only to their intake but also to their chemical structure, food matrix interactions, bioaccessibility and bioavailability [26,27,29].

Human digestion simulation models are essential tools for assessing the nutritional quality of ingredients and foods, as they allow the controlled reproduction of physiological conditions of the gastrointestinal tract [30]. These models enable the investigation of protein and lipid hydrolysis processes, as well as the estimation of the bioaccessibility of inorganic elements and bioactive compounds, providing relevant information on the nutritional and functional potential of unconventional ingredients and foods, such as industrial by-products, which often exhibit complex composition and strong interactions among nutrients [31,32].

Considering the large volume of grape pomace generated by the juice and wine industries in Brazil and the growing interest in the valorization of agro-industrial by-products, it is important to characterize the chemical composition and bioaccessible fractions of these materials. Therefore, this study aimed to evaluate the proximate composition, total phenolic compounds, essential and trace inorganic elements, and the bioaccessible fractions of phenolic compounds and inorganic elements in Brazilian grape pomaces. The combined assessment of mineral composition, trace elements, and in vitro bioaccessibility may contribute to a broader understanding of the nutritional relevance and potential applications of these by-products in food systems.

## 2. Materials and Methods

### 2.1. Materials

For the analytical procedures, purified water obtained by a reverse osmosis system with resistivity lower than 18.2 MΩcm (Gehaka, São Paulo, Brazil) was used. Other reagents included concentrated nitric acid purified using a sub-boiling system (Distillacid, Berghof, Eningen, Germany); hydrochloric acid (37%) and hydrogen peroxide (30%) (Merck, Darmstadt, Germany); α-amylase (10080), pepsin (P6887), bovine bile (B3883), and pancreatin (P7545) from Sigma-Aldrich (St. Louis, MO, USA). All other reagents employed in the preparation of salivary, gastric, and intestinal fluids followed the specifications of the INFOGEST 2.0 protocol [33]. Certified reference materials (CRM) of spinach leaves (NIST 1570a) and peach leaves (ERM-BD 1547) were used to assess the method accuracy [34,35].

For Ca, Na, P, Mg, K, Cu, Fe, Mn, and Zn, analytical calibration curves were prepared from dilutions of certified standard solutions (five calibration points), ranging from 0.041 to 41.0 mg/100 mL for Ca and Na; 0.062 to 62.0 mg/100 mL for P; 0.015 to 14.5 mg/100 mL for Mg; 0.061 to 61.0 mg/100 mL for K; and 0.001 to 1.0 mg/100 mL for Cu, Fe, Mn, and Zn. For trace elements, a multielement standard solution (100 mg/L) and Sb (1000 mg/L) were used. Calibration curves were prepared from dilutions of the certified standard solutions, with five calibration points, ranging from 0.1 to 100 μg/L for Co, As, Mo, Cd, Sb, Ba, Hg, Pb, and Se, and from 5 to 500 μg/L for Al. To correct for matrix and instrumental deviations, certified standard solutions of Ge, Rh, Bi, and Pt at 1000 mg/L (Specsol-Quimlab, Jacareí, Brazil) were used as internal standards at a concentration of 50 μg/L.

#### RawMaterial Collection

Thirteen grape pomace samples were obtained, corresponding to 11 grape varieties. Twelve samples were derived from red grapes as winemaking by-products after fermentation, while one sample corresponded to white grape pomace generated during white wine production (non-fermented). The by-products were donated by wine producers between January and March 2025. Four varieties originated from the interior of São Paulo State (*Maximus*, *Magna*, *Niagara Branca*, and a mixed pomace composed of *Niagara Rosada*, *Isabel*, and *Bordô*), whereas the remaining samples were obtained from the Bento Gonçalves region, Rio Grande do Sul, including *Bourskava*, *Merlot* (2 samples), *Saperavi* (2 samples), *Marselan*, *BRS Carmem*, *Pinot*, and *Cabernet Franc*, totaling 13 samples and 11 varieties. All byproducts were dried in a forced-air oven at 60 °C for 24 h, ground using a mill (IKA, model A11, IKA, Staufen, Germany), sieved through a 0.60 mm mesh, and stored in a freezer until analyses.

### 2.2. Methodologies

#### 2.2.1. Proximate Composition

The grape pomace samples were characterized for moisture and ash contents using the gravimetric method according to AOAC [36]; lipid content by the method proposed by Bligh & Dyer [37]; and protein content was determined by the Kjeldahl [36]. Total carbohydrate content was calculated by the difference method.

#### 2.2.2. Analytical Control

The elements Ca, Cu, Fe, P, Mg, Mn, K, Na, and Zn were determined by inductively coupled plasma optical emission spectrometry (ICP OES) (5100 VDV, Agilent Technologies, Tokyo, Japan), and Al, Co, As, Se, Mo, Cd, Sb, Ba, Hg, and Pb were determined by inductively coupled plasma mass spectrometry (ICP-MS) (iCAP RQ, Thermo Scientific, Bremen, Germany), according to the operating conditions described in Table S1 (Operational parameters of the ICP OES and ICP-MS used for the determination of inorganic elements in grape pomace).

To evaluate the analytical method, certified reference materials of spinach leaves (NIST 1570a) were used for essential elements (Ca, Cu, K, Mg, Mn, Na, P, and Zn) and trace elements (Al, Co, As, and Se), and peach leaves (ERM-BD 1547) were used for Cu and the trace elements Mo, Cd, Sb, Ba, Hg, and Pb. In addition, a grape pomace sample was also used to assess the precision of the analytical method. The figures of merit included linearity, precision, accuracy, and the limits of detection and quantification [34,35].

Linearity was evaluated using analytical curves with five equidistant points of increasing concentration. The analytical curve showed linearity with R^2^ > 0.99 for all elements. Recovery was assessed using certified reference materials, with recovery percentages ranging from 85 to 120% for trace elements and from 86 to 100% for essential elements. Precision, evaluated through seven replicates of a sample, ranged from 2 to 9% for essential elements and from 1 to 15% for trace elements, meeting the coefficient of variation (CV) specifications established by INMETRO [35]. Limits of detection (LOD) and quantification (LOQ) were estimated based on the concentration of the first point of the analytical calibration curve for each analyte, considering the sample preparation dilution factor of 70×. The estimated LOD and LOQ ranges were as follows: Ca and Na (0.8–2.9 mg/100 g), K and P (1.2–4.1 mg/100 g), Mg (0.3–1.0 mg/100 g), Cu, Fe, Mn, and Zn (0.02–0.07 mg/100 g), Al (102–340 µg/L), and Co, As, Mo, Cd, Sb, Ba, Hg, and Pb (2–7 µg/L).

#### 2.2.3. Essential and Trace Elements Determination

For the determinations, the samples were mineralized according to Rebellato [38], with modifications. Approximately 0.3 g of dried sample and 4 mL of HNO_3_ were placed in glass digestion tubes and left to stand overnight. The samples were then digested in a block digester at 110 °C (Tecnal, Piraciacaba, Brazil) for 2 h, followed by cooling to room temperature. Subsequently, 2 mL of H_2_O_2_ was added, and the samples were digested for an additional 2 h at 110 °C. After cooling, the digests were diluted to a final volume of 20 mL with ultrapure water and filtered through a 0.45 µm PTFE membrane filter (Agilent Technologies, Tokyo, Japan). All mineralization procedures were performed in triplicate, including the analytical blank.

For the trace elements, an exposure scenario considered a daily intake of 15 g of grape pomace flour, corresponding to a realistic amount potentially incorporated into functional food products. The estimated exposure to trace elements per eating occasion was calculated according to Equation (1) using the highest concentrations found detected in the analyzed samples and a standard adult body weight (bw) of 60 kg.

(1) Estimated exposure per portion = [conc] × serving size/bw

where estimated exposure per portion = mg/kg bw/day; [conc] = trace element concentration (mg/kg); serving size = daily grape pomace flour intake (kg); and bw = body weight (kg).

The estimated exposure values were compared with Health-Based Guidance Values (HBGV) available in the literature to support the toxicological assessment [39,40,41,42,43,44,45,46].

#### 2.2.4. Total Phenolic Compounds Determination

The phenolic compounds from grape pomace residues were extracted using 0.5 g of sample and 10 mL of a 50% (*v*/*v*) acetone: water solution, in triplicate. The mixture was subjected to an ultrasonic bath (40 kHz, 200 W; Ecosonic Q-5.9 model, Ultrasonic, Indaiatuba, Brazil) for 30 min at room temperature, followed by centrifugation at 4677× *g* for 15 min at 4 °C. The supernatant was filtered and stored at −22 °C, as reported by Infante [47], with modifications.

The total phenolic compounds were determined as described by Singleton [48], adapted for UV/Vis spectrophotometric reading in a 96-well microplate (FLUOstar Omega, BMG Labtech, Ortenberg, Germany). Absorbance was measured at a wavelength of 725 nm. The calibration curve was constructed with seven points using gallic acid, ranging from 20 to 140 mg L^−1^, with R^2^ > 0.99. Results were expressed as mg of gallic acid equivalents (GAE) per 100 g of sample.

#### 2.2.5. Bioaccessibility of Essential Inorganic Elements, Trace Elements, and Phenolic Compounds

Of the 11 grape pomace varieties evaluated, five samples (*Maximus*, *Saperavi_DC*, *Pinot*, *Marselan*, and *Merlot_2*) were selected for the in vitro bioaccessibility assays of essential and trace inorganic elements and phenolic compounds. The selection was based on contrasting compositional characteristics, including total phenolic content and mineral composition, in order to represent distinct nutritional profiles among the analyzed pomaces. This targeted selection strategy enabled the evaluation of samples with different compositional attributes; however, the bioaccessibility results should not be generalized to all grape pomace varieties evaluated in this study.

The in vitro digestion simulation was carried out according to the INFOGEST 2.0 protocol [33], with modifications. The INFOGEST 2.0 protocol was adapted regarding sample mass (approximately 1.3 g of grape pomace and 3.7 g of water) and centrifugation conditions (3500× *g* at 4 °C for 30 min) to accommodate the characteristics of grape pomace matrices. Before the assays, enzymatic activities were evaluated, and gastric lipase was not used. At the end of the intestinal phase, the samples were centrifuged at 3500× *g* and 4 °C for 30 min. The supernatant was transferred to digestion tubes and heated in an oven at 100 °C overnight. Mineralization was then performed as described in Section 2.2.3. All analyses were carried out in triplicate, including the analytical blank.

To evaluate the bioaccessibility of phenolic compounds, aliquots were collected at the end of both the gastric phase and the intestinal phase. The digested samples were stored at −22 °C. The phenolic compounds present in the digested fractions were quantified according to the method described in Section 2.2.5. Bioaccessibility was expressed as the percentage of phenolic compounds released after digestion relative to their initial content in the undigested samples. The content of the element of interest was calculated as follows (Equation (2)):

(2) Bioaccessibility (%) = (content after digestion/content before digestion) × 100

### 2.3. Statistical Analysis

The bioaccessibility results were analyzed using analysis of variance (one-way ANOVA) followed by Tukey’s test (*p* < 0.05) for comparison of means ($\bar{x}$ ± SD), using Statistica software version 7.0 (StatSoft Inc., Tulsa, OK, USA). Principal Component Analysis (PCA) was also performed using Pirouette software version 3.11 (Infometrix, Inc., Bothell, WA, USA).

## 3. Results and Discussion

### 3.1. Proximate Composition and Phenolic Compounds Content

Table 1 presents the results of the proximate composition and total phenolic compounds of the grape pomace samples. Overall, variability was observed among the different grape pomaces for all parameters studied (*p* < 0.05).

The moisture content varied approximately 11% among the different grape pomaces analyzed. This variation is mainly associated with the processing steps used during winemaking, particularly the pressing stage carried out after the fermentation of red grapes or after juice extraction from white grapes (*Niagara*), which does not involve a fermentative process. Due to this variability in moisture content (66.76–77.97%), all residues were dried, and the results were expressed on a dry basis to enable comparison among samples and to ensure the microbiological stability of the resulting flours.

The ash content ranged from 1.32 to 2.87 g/100 g on a dry basis, corresponding to 0.29 to 0.95 g/100 g on a wet basis. This parameter is relevant because it reflects the content of inorganic elements present in the material. According to the literature, fresh grapes present an ash content of approximately 0.5 g/100 g, which is consistent with most of the values obtained in this study [49], except for the mixed pomace sample (0.95 g/100 g), composed of a blend of three grape varieties (*Niagara Rosada*, *Isabel*, and *Bordô*).

The protein content ranged from 6.35 to 13.48 g/100 g (dry basis) and from 1.93 to 4.48 g/100 g (wet basis), with the higher values than those reported by the Brazilian Food Composition Table (TACO) [49] (0.6 g/100 g). It is worth mentioning that the protein content of the white grape residue was lower than that of red grapes, which can be associated with the varieties studied and the fermentation process. The higher protein content is of nutritional relevance, as its application in new food products may contribute to dietary protein intake.

Lipid content ranged from 8.6 to 13.79 g/100 g (dry basis) and from 1.90 to 4.22 g/100 g (wet basis). TACO reports a lipid content of 0.2 g/100 g in raw grapes [49]; this difference can be attributed to the composition of grape pomace, which consists of skins, residual pulp, and seeds, the latter being the fraction in which most lipids are concentrated.

According to the literature, fresh grapes contain approximately 13 g/100 g of carbohydrates. In grape pomace, the carbohydrate content ranged from 15.97 to 25.37 g/100 g on a wet basis, corresponding to 72.18 to 83.60 g/100 g on a dry basis. Based on the proximate composition analysis, the main differences observed among the by-products were the higher ash content in the sample identified as mixed pomace, probably due to the combination of different grape varieties used in wine production, and the *Niagara Branca* grape pomace, which showed the lowest protein content and the highest carbohydrate content, most likely because it did not undergo fermentation, unlike the other grape varieties. Overall, the lipid and protein contents of the grape pomace samples were comparable to values reported by Arcia [22].

Although dietary fiber was not directly determined in the present study, grape pomace is recognized as an important source of fiber, in addition to phenolic compounds and minerals. According to recent literature, grape pomace is mainly composed of skins, seeds, and stalks, containing cellulose, pectins, proteins, lipids, minerals, and other bioactive compounds. Dietary fiber contents ranging from approximately 20 to 50% have been reported, depending on grape variety and processing conditions. This fiber fraction may influence the gastrointestinal bioaccessibility of minerals and phenolic compounds through physicochemical interactions involving fiber components and tannins, potentially affecting compound release and absorption [24,28,50].

Regarding total phenolic compounds, the content ranged from 566.73 to 1842.78 mg GAE/100 g among the grape pomace samples. As shown in Table 1, the by-products from the grape varieties *Merlot_2*, *Saperavi_DC*, *Maximus*, and *Pinot* exhibited the highest total phenolic contents (TPC), while the varieties *Magna*, *Niagara Branca*, *Cabernet Franc*, and mixed pomace showed the lowest values. This variation may be related not only to grape variety, but also to geographical origin and harvest period. Although obtained from the same wine producer, the *Merlot* and *Merlot_2* samples were harvested and processed at different times in Bento Gonçalves (RS), while the varieties *Magna*, *Niagara Branca*, and mixed pomace were produced in the state of São Paulo.

Phenolic compounds are of considerable interest to Brazilian industries, including the pharmaceutical, food, and cosmetic sectors. As can be seen in Table 1, the *Saperavi_DC* variety showed the highest TPC content, with 1842.78 mg GAE/100 g on a dry basis. Arcia [22] evaluated the composition of different fruit pomaces, including by-products from the production of wine from the Tannat variety (Uruguay). Those authors reported values ranging from 1080 to 1200 mg GAE/100 g (dry basis), which are similar to some of the varieties evaluated in the present study. On the other hand, Martinović [51] used a 50% aqueous ethanol extraction solution and found higher total phenolic contents in grape pomace from the *Cabernet Sauvignon* variety (Croatia, 2018 harvest). These data show that not only extraction conditions, but also agronomic, geographical, and harvest conditions have a strong influence on the phenolic compound content of grape pomace.

The total phenolic contents of the grape pomaces (566.73 to 1842.78 mg GAE/100 g) in this study indicate that this by-product of the wine industry has high potential as a source of polyphenols. When incorporated into food formulations, these compounds may contribute to the intake of dietary polyphenols, which have been associated in the literature with beneficial physiological effects [24]. It is important to note that the Folin–Ciocalteu assay provides an estimation of total reducing capacity and may be influenced by non-phenolic reducing compounds. In addition, detailed phenolic profiling was not performed in the present study. Therefore, the results should be interpreted as an overall estimation of total phenolic-related compounds rather than a specific characterization of individual phenolics.

### 3.2. Quantification of Essential Inorganic Elements

Table 2 presents the composition of essential inorganic elements (Ca, Cu, Fe, K, Mg, Mn, Na, P, and Zn, expressed as mg/100 g on a dry basis) in the different grape by-products. In general, most of the essential elements evaluated in the different grape by-products showed significant differences among them (*p* < 0.05).

Among the grape varieties studied, the *Niagara Branca* variety exhibited the lowest levels for most of the elements analyzed, except for Mn (2.51). The by-product of the *Bourskava* variety showed the highest contents of Ca (327.71) and Cu (27.26). The *Saperavi_DC* and *Merlot* varieties also presented high Ca contents (336.32 and 332.14, respectively), with no significant difference compared to the *Bourskava* variety.

The mixed pomace, composed of the *Niagara Rosada*, *Isabel*, and *Bordô* grape varieties, exhibited the highest contents of Fe (4.44), K (3062.70), and Zn (3.48), whereas the *Maximus* variety presented the highest levels of Mg (117.08) and P (315.20). In turn, the *Pinot* and *Cabernet Franc* varieties showed the highest Na (6.73) and Mn (8.27) contents, respectively. As observed for Ca, both K and Mg are macroelements absorbed from the soil by plants and are therefore present at high concentrations in grape-derived products [52].

Mohamed Ahmed [53] investigated the chemical composition, bioactive compounds, minerals, and fatty acid composition of grape pomace from 10 varieties, including seeds and skins from different grape varieties, originating from the provinces of Mersin and Konya, Turkey. The authors found that Fe (28.86–54.68 mg/g), P (31.57–15.61 mg/g), Zn (22.51–12.64 mg/g), and K (27.18–14.48 mg/g) were the main minerals present in the varieties studied. In contrast, Machado [24] reported that red grape pomace from the Portuguese cultivar Vinhão proved to be an excellent source of K (6051.94), Ca (946.41), Fe (36.38), and Mn (4.30), with results expressed in mg/100 g on a dry basis. Barriga-Sánchez [54] evaluated the chemical composition and the mineral contents of Ca, Fe, Cu, and Zn in Burgundy black grapes, as well as in their pomace and seeds. The authors observed that the seeds presented the highest Ca content (137.24 mg/100 g), whereas the pomace showed higher amounts of Fe (12.16 mg/100 g) and Cu (1.18 mg/100 g). Regarding Zn content, no significant difference was reported between the pomace (0.86 mg/100 g) and the seeds (0.81 mg/100 g).

The differences observed between the present study and the literature can be attributed to grape cultivar varieties, geographic location, climate, seasonal influences, chemical composition, including soil composition, irrigation systems, stage of maturation, viticulture practices, and pressing efficiency during vinification, as reported by Barriga-Sánchez and Chikwanha [54,55]. It is important to note that no soil, climate, or vineyard management analyses were performed in the present study. Therefore, the relationships discussed between mineral composition and environmental or cultivation factors should be interpreted only as possible explanations supported by previous literature.

### 3.3. Total Content of Trace Inorganic Elements

Ten trace elements were evaluated in the different grape by-products, and the results are presented in Table 3. Overall, significant differences (*p* < 0.05) were observed for the trace elements of most of the varieties studied.

Among the elements evaluated, Se and Mo stand out as they are considered essential to health when ingested at appropriate concentrations [56,57,58]. The grape pomace from the *Niagara Branca* variety presented the lowest Se content (14.69 µg/kg) and the highest Mo content (399.44 µg/kg). In contrast, the pomace from the *Saperavi* variety showed the highest Se content (1269.88 µg/kg), while the *Merlot* variety presented the lowest Mo content (44.27 µg/kg). In general, high selenium levels in foods are associated with cultivation in selenium-rich soils or with the addition of isolated salts [49], which may explain the values observed for the *Saperavi* pomace.

In turn, the highest contents of As, Cd, Pb, and Hg were observed in the residues of the *Saperavi*, *Maximus*, and mixed pomace varieties, whereas the lowest contents were found in the residues of the *Magna* and mixed pomace, *BRS Carmem*, *Cabernet Franc*, and *Maximus* varieties, respectively. It is worth noting that residues from different grape varieties presented values below the limit of quantification for the contaminants Hg, Cd, and Pb (4 µg/kg).

The elements As, Cd, and Pb have maximum tolerable limits (MTL) established for different foods, according to Normative Instruction No. 160/2022 [59]. For fresh berries, such as fresh grapes, the maximum limits are 0.30 mg/kg for total As, 0.05 mg/kg for Cd, and 0.2 mg/kg for Pb. Regarding mercury, although MTLs are defined for several foods, there is no specific reference for fresh berries. Thus, despite the quantification of these elements in residues from some grape varieties, the levels found do not exceed the established limits and therefore do not pose a health risk.

Although no maximum limits have been established for Al, Mo, Sb, and Ba in fresh berry fruits, the intake of these elements should be considered with caution, given the potential risks associated with exposure to elevated concentrations. The mixed pomace (*Niagara Rosada*, *Isabel*, and *Bordô*) presented the highest Al content (234.32 mg/kg), whereas the *Niagara Branca* residue showed the lowest value (17.57 mg/kg). The *Merlot* residue exhibited the highest contents of Sb (138.71 µg/kg) and Ba (50,480.36 µg/kg), as well as the lowest Mo content (44.27 µg/kg), when compared with the other varieties. In contrast, the *Niagara Branca* residue presented the highest Mo content (399.43 µg/kg), while the *Pinot* and *Marselan* varieties showed the lowest contents of Sb (<LOQ, 4 µg/kg) and Ba (2099.54 µg/kg), respectively.

Studies evaluating the mineral composition and availability of grape pomace have demonstrated that the distribution and mobility of trace elements may vary according to grape variety, processing conditions, and the anatomical fractions of the residue. Pérez Cid [60] reported differences in metal accumulation between grape skins and seeds and observed distinct mobility patterns for several elements, highlighting the importance of evaluating not only total concentrations but also element availability when assessing the potential applications and safety of grape pomace-derived products. These observations are consistent with the present study, in which considerable variations were also observed among the analyzed grape pomace varieties, particularly regarding the bioaccessible fractions of minerals.

The exposure per serving was estimated for each trace element using the highest concentrations detected among the analyzed samples, considering a daily intake of 15 g of grape pomace flour, corresponding to a realistic amount potentially incorporated into functional food products, and a standard adult body weight of 60 kg. To characterize the potential risk associated with trace element intake, the highest estimated exposure values (mg/kg bw day) were compared with the reference values presented in Table 4 for each element.

It is important to note that serving size directly influences the estimated exposure values and that grape pomace flour is not the sole dietary source of these trace elements. In addition, the present assessment considered only the estimated exposure per serving and its percentage contribution to the Health-Based Guidance Values (HBGV). Therefore, it does not represent the overall dietary contribution of grape pomace consumption within the total diet.

The highest estimated exposure values were observed for Al and Co, reaching 0.05858 and 0.000625 mg/kg bw/day for adults, respectively, in the Mixed and *Magna* samples. These values corresponded to 20.5% of the Provisional Tolerable Weekly Intake (PTWI) established for Al and 39% of the HBGV established for Co [40,45]. For Ba and Hg, the highest estimated exposure values were 0.01262 mg/kg bw day and 0.000012925 mg/kg bw day, respectively, observed in the *Merlot* pomace and Mixed samples. These exposure levels corresponded to 6.3% of the Tolerable Daily Intake (TDI) established for Ba and 5.7% of the Provisional Tolerable Weekly Intake (PTWI) established for Hg, indicating relatively low contributions to the respective toxicological reference values [41,46].

The estimated exposure values for the remaining trace elements represented less than 1% of the respective HBGV established in the literature, indicating low toxicological concern under the hypothetical consumption scenario considered in this study [39,41,42,43,44,45].

The potential application of grape pomace as a sustainable ingredient for human nutrition has also been reported in previous studies. Pereira [61] evaluating different grape pomace varieties, highlighted that this agro-industrial by-product represents a promising source of nutrients and bioactive compounds for food applications, while emphasizing the importance of monitoring trace and potentially toxic elements to ensure food safety and compliance with regulatory limits.

### 3.4. Bioaccessibility of Essential and Trace Inorganic Elements and Phenolic Compounds

Table 5 and Table 6 present the results of the bioaccessibility studies for essential inorganic elements, phenolic compounds, and trace elements, respectively. In general, only grape pomace from the *Saperavi_DC* variety showed a quantifiable bioaccessible fraction of Ca (5.8 mg/100 g); although the total Ca content of this sample was 336 mg/100 g, the bioaccessible percentage was only 1.7%. A similar behavior was observed for the elements Fe and Zn: although it was possible to quantify the total content of these elements in the residues of the selected varieties, the bioaccessible fractions were below the LOQ. These results may be associated with the high concentration of phenolic compounds present in grape pomace, especially flavonoids and tannins, which contain catechol and galloyl groups [62]. At the physiological pH of the intestinal tract, the deprotonation of these phenolic groups favors coordination with divalent metal ions, inducing the formation of poorly soluble complexes and reducing the bioaccessibility of the mineral [63]. Furthermore, the reduced bioaccessibility of calcium observed in some samples may be associated with its interactions with other components of the food matrix. Calcium is known to strongly interact with dietary fibers, particularly pectins, forming insoluble complexes that limit its release during gastrointestinal digestion [62].

The residues of the *Pinot* and *Merlot_2* varieties stood out for presenting the highest bioaccessible percentages of Cu (13%), whereas the *Saperavi_DC* and *Marselan* varieties showed the lowest percentages, 5.5% and 6.8%, respectively.

Regarding the element Mg, although the bioaccessible percentage ranged from 27 to 30%, no significant difference (*p* < 0.05) was observed among the residues studied. In general, the polysaccharide profile of the fibrous plant matrix is capable of negatively interfering with the bioaccessibility of minerals such as magnesium. In this context, the negative charge of the carbohydrate may lead to the formation of complexes through electrostatic interaction which, in the digestive tract, is responsible for decreasing the solubilization of the mineral [64].

For the elements Mn and P, the residues of the *Saperavi_DC* and *Maximus* varieties showed the highest bioaccessible percentages (14% and 26%, respectively). The lowest bioaccessible percentage of Mn was observed for the *Merlot_2* variety; for the *Saperavi_DC*, *Pinot*, *Marselan*, and *Merlot_2* varieties, no significant differences (*p* > 0.05) were observed among the bioaccessible percentages of phosphorus (approximately 20%). Brazilian commercial flours formulated from grape by-products have already shown higher bioaccessibility percentages than those reported in the present study, based on in vitro simulation of adult gastrointestinal digestion. In that study, the authors reported approximate proportions of 20% bioaccessible calcium, 60–70% bioaccessible magnesium, and 2.5% bioaccessible iron. However, it is important to emphasize that commercial flour formulations may contain different proportions of edible and non-edible grape parts, which would directly impact the total mineral composition, as well as the presence of antinutritional compounds such as tannins, phytates, and oxalates, which are capable of interacting with inorganic elements, forming insoluble complexes during gastrointestinal digestion [65], impairing comparisons with the present study.

The bioaccessible fraction of phenolic compounds in the grape by-product varieties was evaluated during the gastric and enteric phases (Table 5). Except for the *Marselan* variety, significant differences were observed between the gastrointestinal phases for all other varieties (*p* < 0.05). The bioaccessible percentage in the gastric phase (GP) ranged from 5.95% (*Merlot_2*) to 13.79% (*Pinot*), whereas in the enteric phase the values ranged from 6.98% (*Marselan*) to 19.18% (*Pinot*). The bioaccessibility of grape phenolic compounds, such as anthocyanins and procyanidins, may be modulated by the physical characteristics of the plant material, as well as by interactions with proteins and digestive enzymes, and by the physiological conditions. Interactions between phenolic compounds and proteins of the food matrix, for example, are capable of impacting protein conformation, charge distribution, and protein digestibility, directly interfering with the protein digestion process and, consequently, with the bioaccessibility of phenolic compounds [25]. Gastric and/or intestinal pH conditions are also responsible for the stability of certain phenolic compounds, leading to structural transformations and copigmentation phenomena, and interfering with their bioaccessibility.

According to Morales-Ovando [66], the low bioaccessible fraction of phenolic compounds can also be attributed to multiple concurrent phenomena, including oxidative, acidic, and enzymatic degradation; polymerization into higher molecular weight compounds; precipitation into the non-bioaccessible fraction; and transformation into non-quantifiable molecules. Furthermore, phenolic compounds may undergo complexation reactions with proteins, carbohydrates, and mineral ions, which can limit their release and availability.

In the oral phase, rapid diffusion of phenolic compounds into the solution is observed, accompanied by the immediate release of these compounds from the surface of the grape by-product. This diffusion intensifies during the gastric phase (GP) and, with the reduction in pH from neutral to acidic, the solution acquires a reddish coloration due to the presence of anthocyanins in the grape pomace residue. In the intestinal phase (IP), the solution presents a brown coloration, resulting from pigment degradation. The intestine represents the main site of phenolic compound metabolism, as it provides greater opportunity for absorption and allows the occurrence of bioactive properties, such as anti-inflammatory, anticancer, and antimicrobial activities, among others [51].

The high content of total phenolic compounds in grape by-products prior to digestion is mainly associated with the presence of oligomeric and polymeric proanthocyanidins, which represent the most abundant phenolic constituents of the antioxidant fraction of grapes [67]. Part of these proanthocyanidins is bound to the fiber matrix through weak interactions, which allows their extraction and quantification [68,69]. After gastrointestinal digestion, however, a significant reduction in total phenolic content and antioxidant activity is observed, mainly attributed to anthocyanin degradation. Studies have shown expressive qualitative and quantitative reductions in the phenolic profile of grape pomace, especially for anthocyanin compounds, whose concentration may decrease by nearly fivefold [22,32].

In general, phenolic compounds exhibit moderate to high solubility in water, and their bioaccessibility is directly associated with the efficiency of compound release from the food matrix and its subsequent dissolution in the aqueous phase during digestion. However, phenolic compounds are commonly structured in the form of esters, glycosides, or polymers, and are not directly absorbable by the human body. In this context, enzymatic action and intestinal microbiota metabolism are responsible for promoting the conversion of these derivatives into glucuronated, methylated, or sulfonated metabolites, generating derivatives with greater absorption potential [70,71].

The bioaccessibility of trace inorganic elements was also evaluated to estimate the available fraction, and the results are presented in Table 6. Although higher total Al levels were observed in the residues, the bioaccessible percentage ranged from 2.3% (*Maximus*) to 13% (*Merlot_2*). Among the varieties, the *Maximus* grape residue exhibited a high bioaccessible As percentage (88%), similar to that observed for the *Merlot*_2 variety (66%). Except for the *Merlot_2* variety, the other varieties presented bioaccessible percentages ranging from 29 to 72% for Co. Regarding the element Ba, bioaccessibility ranged from 1.1 to 9%, except for the *Marselan* variety, which presented results below the LOQ for this element.

Haas [52] investigated the bioaccessibility of phenolic compounds, macroelements, and microelements in the sediment of grape juice from the *Bordô* and *Isabel* red grape varieties. The authors reported that Ni and Pb were not detected in the samples and that the concentrations of Al, Sr, and Cu were below the detection limit after simulated digestion. In addition, those authors reported that it is worth noting that metals and semimetals are potentially toxic, leading to acute symptoms in the kidneys, liver, heart, vascular system, and immune system [72].

The growing interest in grape pomace valorization has also been highlighted in review studies. Antonić [73], in a systematic review and meta-analysis, reported that grape pomace has been successfully applied in the fortification of different food products, including bakery, dairy, meat, and fish products, mainly due to its high contents of polyphenols and dietary fiber. According to the authors, the incorporation of grape pomace generally improved the nutritional quality and oxidative stability of fortified foods, reinforcing the potential of this agro-industrial by-product as a sustainable ingredient for food applications.

Although previous studies have investigated the bioaccessibility of phenolic compounds in grape pomace, limited information is available regarding the combined evaluation of inorganic elements and phenolic compounds under simulated gastrointestinal digestion, particularly considering the grape species analyzed in this study. This gap is relevant given that grape pomace is a byproduct of recognized nutritional and functional importance. Therefore, the results presented here contribute to expanding current knowledge on the bioaccessible fraction of these compounds, providing support for future research and potential applications in food science and nutrition.

### 3.5. Principal Component Analysis (PCA)

**Figure 1:**
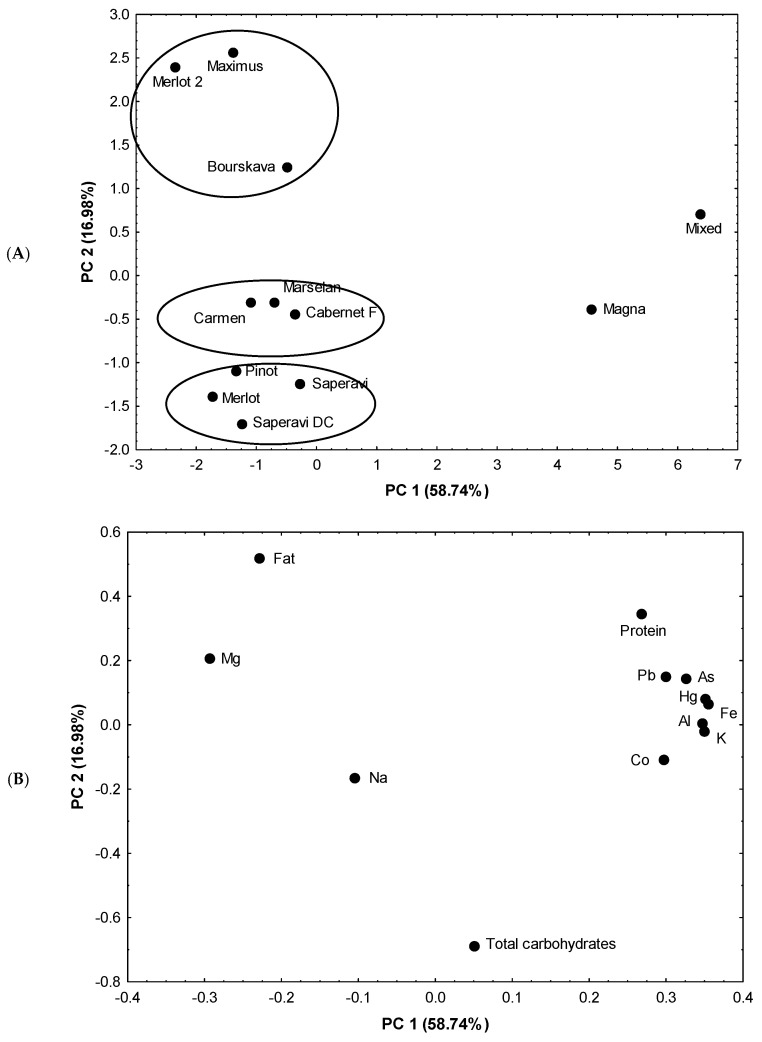
Score (**A**) and loading (**B**) plots.

To verify possible correlations among the parameters proximate composition, essential and trace inorganic elements, and total phenolic compounds, a Principal Component Analysis (PCA) was performed. The PCA was constructed using the results of fermented grape by-products and the 25 variables studied (total solids, ash, proteins, lipids, carbohydrates, total phenolic compounds, essential and trace inorganic elements), resulting in a matrix of dimension 12 × 25, representing 300 assays. Data were autoscaled, and the variables were selected considering their modeling power (above 0.35), resulting in a matrix of dimension 12 × 12. The model accounted for 85.82% of the variance, corresponding to the sum of the first (PC1), second (PC2), and third (PC3) principal components. The graphical representation of the principal components is shown in Figure 1, with 75.72% of the variance explained by PC1 and PC2 (score and loading plots).

In the score plot (A) and the loading plot (B), the grape by-products were distributed into three distinct groups, and two samples were not classified, remaining isolated. One group was formed by the by-products of the *Maximus*, *Merlot_2*, and *Bourskava* grape varieties, which were correlated due to presenting the highest lipid contents. A second group was composed of the *Pinot*, *Merlot*, *Saperavi* and *Saperavi_DC* grape by-products, which were grouped due to their higher contents of total carbohydrates, as well as the essential inorganic element Mg.

The third group, composed of the by-products of the *Cabernet Franc*, *BRS Carmen*, and *Marselan* grape varieties, was grouped due to presenting a similar Na, and Mg composition. The *Magna* variety stood out for presenting a high correlation with Co content. The mixed pomace was separated from the others due to presenting higher contents of proteins, ash, K, Fe, and Zn, as well as the trace inorganic elements Co, Mo, Pb, As, Hg, Al, and Sb.

In general, cultivars are contaminated by soil, water, or the atmosphere, or may also be contaminated along their production chain. The presence of toxic inorganic elements is of concern due to their cumulative toxicity and their potential non-carcinogenic and/or carcinogenic effects, which may adversely impact human health [74,75,76].

Aluminum and arsenic may be part of the composition of grape-derived products as a consequence of the use of herbicides and insecticides in grape production [55,77,78]. The use of sodium arsenite-based pesticides (NaAsO_2_) was previously employed in viticulture to protect grapevines against fungi that cause significant damage to fruit production and quality. However, due to its high toxicity and carcinogenic nature, its use has been banned in many regions, including the European Union, the United States, and Brazil [78,79,80]. With regard to aluminum, it is known that soil contains on average 10% total Al, and under acidic conditions this element can be solubilized and absorbed by plants [77]. In Brazil, particularly in the state of Rio Grande do Sul, most vineyards are located in acidic soils with high Al content [81]. Although Al is not considered an essential element for the human body, there are several possible routes of ingestion, such as food, additives, antacids, and water, among others. Its natural presence in foods is low; however, authors have already associated Al intake with neurological diseases and sclerosis [82]. In turn, although Cu is not produced or synthesized in the body, a small daily intake is required to meet its metabolic functions, whereas excessive consumption may present toxic effects [83].

Concerning arsenic (As), the main route of chronic exposure for humans occurs via dietary intake. Therefore, before consuming a given by-product, it is essential to know its chemical composition and inorganic element content, as well as to estimate the availability of these compounds for utilization by the body, to assess the feasibility of its use in human nutrition.

## 4. Conclusions

This study evaluated the proximate composition, total phenolic compounds, essential and trace elements, and the in vitro bioaccessibility of selected compounds in grape pomaces produced in São Paulo and Rio Grande do Sul, Brazil. The results demonstrated considerable variation among the evaluated pomaces regarding phenolic compounds and mineral composition. Although the pomaces exhibited relevant total concentrations of phenolic compounds and essential elements, the bioaccessible fractions were generally low to moderate, particularly for Ca, Fe, and Zn. These findings highlight the importance of considering bioaccessibility in addition to total composition when assessing the nutritional relevance of grape pomace.

Potentially toxic elements such as As, Cd, Pb, and Hg were detected at concentrations below the maximum limits established by Brazilian legislation. The exposure assessment based on a daily intake of 15 g of grape pomace flour indicated that the estimated exposure values for the evaluated trace elements remained below the respective HBGV. Although Al and Co showed the highest contribution percentages relative to the health-based guidance values, none of the evaluated samples exceeded toxicological reference limits under the proposed consumption scenario.

The observed compositional differences among samples may be associated with factors such as grape variety and cultivation conditions, although these variables were not directly investigated. Overall, the present findings contribute to the chemical characterization of Brazilian grape pomaces and support further studies regarding their application as value-added agro-industrial by-products in food formulations.

## Figures and Tables

**Table 1:** Proximate composition and total phenolic compounds content of the grape pomace samples.

Grape Pomace Varieties	Solids g/100 g	Ash g/100 g	Proteins g/100 g	Lipids g/100 g	Carbohydrates g/100 g	Total Phenolics mg GAE/100 g
**Boursuaka**	23.38 ± 1.05 ef	1.66 ± 0.04 cde	12.32 ± 0.57 abc	12.21 ± 0.44 c	73.80 ± 0.71 de	1114.43 ± 0.85 cd
**Merlot_2**	22.14 ± 2.03 f	1.32 ± 0.07 g	11.86 ± 0.98 bc	14.71 ± 0.33 a	72.11 ± 1.21 f	1475.74 ± 0.76 b
**Saperavi_Dc**	26.95 ± 0.10 cd	1.61 ± 0.04 cdef	10.69 ± 0.21 cd	9.87 ± 0.13 ef	77.83 ± 0.53 b	1842.78 ± 1.01 a
**Marselan**	26.81 ± 0.74 de	1.83 ± 0.03 bc	12.46 ± 0.29 ab	9.43 ± 0.05 fg	76.27 ± 0.37 bc	1298.16 ± 1.31 bc
**Maximus**	30.59 ± 0.92 ab	1.95 ± 0.10 b	12.08 ± 0.48 bc	13.79 ± 0.36 b	72.18 ± 0.60 ef	1595.31 ± 2.42 ab
**Niagara Br**	30.35 ± 0.46 abc	1.39 ± 0.13 fg	6.35 ± 0.06 e	8.66 ± 0.51 g	83.60 ± 0.28 a	649.80 ± 0.49 ef
**BRS Carmem**	27.11 ± 0.90 bcd	1.56 ± 0.04 def	11.52 ± 0.06 bcd	10.79 ± 0.14 de	76.13 ± 0.08 bc	1090.33 ± 1.48 cd
**Merlot**	26.99 ± 1.40 cd	1.68 ± 0.08 cde	10.21 ± 0.05 d	10.82 ± 0.11 d	77.28 ± 0.08 b	1054.14 ± 0.89 cd
**Pinot**	25.78 ± 0.28 de	1.69 ± 0.10 cd	11.73 ± 0.20 bc	9.59 ± 0.07 f	77.00 ± 0.23 b	1535.81 ± 3.42 ab
**Magna**	22.03 ± 0.68 f	1.69 ± 0.09 cd	12.67 ± 0.47 ab	8.60 ± 0.40 g	77.04 ± 0.02 b	446.10 ± 0.33 f
**Saperavi**	24.24 ± 1.58 def	1.44 ± 0.07 efg	11.68 ± 0.17 bc	9.87 ± 0.56 f	77.01 ± 0.48 b	647.79 ± 0.70 ef
**Cabernet Franc**	24.99 ± 2.33 def	1.70 ± 0.08 bcd	11.31 ± 0.85 bcd	10.91 ± 0.36 d	76.07 ± 0.85 bc	938.85 ± 0.41 de
Mixed *	33.24 ± 0.16 a	2.87 ± 0.13 ab	13.48 ± 0.32 a	8.60 ± 0.27 g	75.04 ± 0.31 cd	566.73 ± 0.46 f

Mean ± SD (*n* = 3), on a dry basis. * Mixed pomace: A blend composed of grape pomace from the *Niagara* Rosada, *Isabel*, and *Bordô* varieties. Mean values with different lowercase letters in the same column indicate significant difference (*p* < 0.05) among samples, as determined by one-way ANOVA and Tukey test at 95% of confidence.

**Table 2:** Total content of essential inorganic elements.

Grape Pomace Varieties	Ca	Cu	Fe	K	Mg	Mn	Na	P	Zn
**Bourskava**	327.71 ± 4.05 a	27.26 ± 0.59 a	5.15 ± 0.26 gh	2237.26 ± 31.15 d	86.04 ± 1.40 fg	2.19 ± 0.04 i	4.20 ± 0.05 c	266.56 ± 4.74 e	1.20 ± 0.09 fg
**Merlot_2**	303.32 ± 4.63 b	9.62 ± 0.09 b	5.01 ± 0.13 h	1770.77 ± 24.63 i	105.62 ± 1.15 c	2.75 ± 0.03 g	3.54 ± 0.26 d	270.58 ± 3.12 de	2.03 ± 0.04 b
**Saperavi_Dc**	336.32 ± 8.52 a	1.55 ± 0.03 h	5.54 ± 0.15 fgh	2002.41 ± 43.54 ef	86.72 ± 1.44 f	6.16 ± 0.11 b	3.25 ± 0.22 de	291.97 ± 4.48 bc	1.57 ± 0.11 cde
**Marselan**	206.90 ± 5.51 de	1.80 ± 0.01 h	5.98 ± 0.16 defg	1764.58 ± 14.97 i	94.07 ± 1.12 d	5.18 ± 0.07 c	2.54 ± 0.09 f	299.78 ± 4.75 b	1.75 ± 0.08 cd
**Maximus**	250.82 ± 3.87 c	1.22 ± 0.04 hi	6.87 ± 0.14 cd	1971.97 ± 29.00 fg	117.08 ± 0.95 a	2.68 ± 0.04 gh	2.65 ± 0.26 f	315.20 ± 2.50 a	0.63 ± 0.01 h
**Niagara**	157.66 ± 3.34 g	0.87 ± 0.02 i	2.70 ± 0.12 i	1446.75 ± 29.03 j	63.61 ± 1.48 i	2.51 ± 0.05 h	1.50 ± 0.13 g	156.54 ± 3.64 g	0.10 ± 0.02 i
**BRS Carmem**	205.36 ± 1.91 de	4.04 ± 0.03 f	7.09 ± 0.60 c	1861.94 ± 8.89 hi	89.29 ± 0.38 ef	1.68 ± 0.01 jk	2.62 ± 0.18 f	247.43 ± 1.24 f	1.03 ± 0.07 g
**Merlot**	332.14 ± 10.26 a	5.22 ± 0.13 e	5.37 ± 0.11 gh	1888.25 ± 47.28 gh	94.06 ± 2.83 d	4.02 ± 0.14 e	4.73 ± 0.10 b	279.66 ± 9.29 cd	1.06 ± 0.05 g
**Pinot**	217.31 ± 0.59 d	8.31 ± 0.07 c	5.82 ± 0.21 efgh	2255.83 ± 16.14 cd	111.91 ± 1.20 b	4.93 ± 0.08 d	6.73 ± 0.19 a	300.52 ± 1.54 b	1.52 ± 0.07 de
**Magna**	204.98 ± 2.55 de	2.59 ± 0.08 g	9.67 ± 0.26 b	2941.02 ± 18.53 b	77.61 ± 0.26 h	3.14 ± 0.02 f	2.78 ± 0.22 ef	264.76 ± 1.68 e	1.21 ± 0.07 fg
**Saperavi**	187.47 ± 2.43 f	4.21 ± 0.02 f	6.47 ± 0.08 cde	2078.31 ± 3.16 e	82.55 ± 0.79 g	1.57 ± 0.01 k	2.48 ± 0.09 f	235.40 ± 1.53 f	1.34 ± 0.08 ef
**Cabernet Franc**	303.93 ± 6.10 b	6.38 ± 0.10 d	6.41 ± 0.36 cdef	2337.32 ± 18.05 c	90.83 ± 1.38 de	8.27 ± 0.16 a	4.54 ± 0.29 bc	266.56 ± 3.94 e	1.81 ± 0.19 bc
Mixed *	192.18 ± 5.35 ef	4.44 ± 0.09 f	13.50 ± 0.73 a	3062.70 ± 87.84 a	61.11 ± 1.32 i	1.80 ± 0.04 j	3.01 ± 0.18 ef	241.76 ± 5.79 f	3.48 ± 0.16 a

Mean ± standard deviation (*n* = 3) in grape pomace. Results expressed in mg/100 g, on a dry basis. * Mixed pomace: A blend composed of grape pomace from the *Niagara Rosada*, *Isabel*, and *Bordô* varieties. Mean values with different lowercase letters in the same column indicate significant difference (*p* < 0.05) among samples, as determined by one-way ANOVA and Tukey test at 95% of confidence.

**Table 3:** Total content (µg/kg) of trace elements in grape pomace.

Grape Pomace Varieties	Al	Co	As	Se	Mo	Cd	Sb	Ba	Hg	Pb
**Bourskava**	27,354.5 ± 3323.9 gh	ND	40.22 ± 9.74 b	16.11 ± 3.77 d	208.21 ± 26.38 c	ND	47.95 ± 6.80 cde	4178.4 ± 1002.93 gh	ND	171.87 ± 63.53 ab
**Merlot_2**	23,150.3 ± 2151.9 gh	ND	43.29 ± 6.88 b	19.01 ± 2.23 d	167.88 ± 17.45 d	ND	52.02 ± 8.15 cd	22,559.2 ± 493.14 d	ND	ND
**Saperavi_Dc**	76,600.7 ± 5926.2 d	48.0 ± 6.3 d	24.09 ± 3.85 bc	1033.75 ± 206.29 b	123.85 ± 4.49 e	ND	25.51 ± 1.32 ef	3823.6 ± 4.48 gh	ND	ND
**Marselan**	92,845.5 ± 1707.1 cd	53.0 ± 8.3 d	26.87 ± 6.93 bc	1234.07 ± 76.42 a	123.04 ± 14.86 e	ND	89.82 ± 20.84 b	2099.5 ± 481.06 h	ND	ND
**Maximus**	73,261.8 ± 3673.9 de	64.42 ± 7.41 d	43.02 ± 4.48 b	197.02 ± 39.67 c	278.90 ± 10.17 b	10.45 ± 0.24 a	49.47 ± 6.84 cde	14,168.3 ± 114.41 f	6.84 ± 1.49 cd	ND
**Niagara**	17,570.1 ± 1442.8 h	ND	30.05 ± 3.78 bc	14.68 ± 2.59 d	399.44 ± 26.09 a	ND	56.89 ± 10.3 cd	4544.55 ± 342.96 gh	ND	ND
**BRS Carmem**	46,680.7 ± 3363.9 f	ND	30.96 ± 7.92 bc	20.74 ± 3.88 d	88.54 ± 3.68 ef	8.55 ± 1.47 b	49.89 ± 10.92 cde	21,225.5 ± 479.36 d	ND	ND
**Merlot**	32,015.1 ± 1264.5 gh	ND	43.42 ± 2.13 b	36.86 ± 1.06 cd	44.27 ± 0.78 f	ND	138.71 ± 24.83 a	50,480.4 ± 3372.92 a	9.10 ± 0.2 c	ND
**Pinot**	54,268.5 ± 1179.9 ef	29.62 ± 10.96 d	35.27 ± 1.89 bc	57.37 ± 4.26 cd	118.97 ± 8.93 e	ND	ND	45,488.7 ± 1004.64 b	ND	ND
**Magna**	163,128.0 ± 2784.4 b	2501.73 ± 128.39 a	114.06 ± 10.45 a	92.44 ± 19.48 cd	238.32 ± 15.38 bc	ND	65.56 ± 2.95 bc	6890.8 ± 406.71 g	39.46 ± 2.84 b	134.37 ± 14.95 bc
**Saperavi**	38,987.8 ± 3128.2 fg	2208.34 ± 128.38 b	14.18 ± 1.42 c	1269.88 ± 32.6 a	71.50 ± 5.36 f	ND	34.77 ± 0.16 de	20,511.1 ± 472.83 de	ND	ND
**Cabernet Franc**	100,992.0 ± 6077.8 c	148.01 ± 37.80 d	33.81 ± 8.17 bc	45.80 ± 4.00 cd	256.89 ± 16.36 b	ND	36.86 ± 8.59 de	33,978.3 ± 321.22 c	ND	99.23 ± 3.55 c
Mixed *	234,321.0 ± 13,412.9 a	1937.81 ± 153.72 c	99.74 ± 17.16 a	80.00 ± 5.94 cd	242.78 ± 9.46 bc	ND	131.15 ± 1.60 a	17,521.4 ± 430.43 e	51.70 ± 2.03 a	216.84 ± 12.87 a

Mean ± standard deviation (*n* = 3). Results expressed in µg/kg, on a dry basis. * Mixed pomace: composed of grapes from the *Niagara* Rosada, *Isabel*, and *Bordô* varieties. ND = not detected; result below the limit of quantification. Limits of quantification (LOQ): Co, As, Mo, Cd, Sb, Ba, Hg, and Pb = 7 µg/kg; Al = 340 µg/kg. Mean values with different lowercase letters in the same column indicate significant difference (*p* < 0.05) among samples, as determined by one-way ANOVA and Tukey test at 95% of confidence.

**Table 4:** Exposure per portion values of trace inorganic elements (mg/kg bw), considering the recommended portion (15 g) for adults (body weight, bw = 60 kg).

Elements	Reference Value and Health-Based Guidance Values Established (HBGV)		Highest Exposure per Portion (mg/kg bw Day)	Grape Pomace
Al	PTWI	2 mg/kg bw [45]	0.05858	*Mixed*
Co	HBGV	0.0016 mg/kg bw [40]	0.000625	*Magna*
As	BMDL_0.5_	i-As 0.003 mg/kg bw [39]	0.0000275	*Magna*
Mo	UL	0.6 mg [43]	0.00009975	*Niagara*
Cd	PTMI	0.025 mg/kg bw [45]	0.0000025	*Maximus*
Sb	TDI	0.006 mg/kg bw [41]	0.000034675	*Merlot*
Ba	TDI	0.2 mg/kg bw [41]	0.01262	*Merlot*
Hg	PTWI	0.0016 mg/kg bw [46]	0.000012925	*Mixed*
Pb	BMDL_10_	0.015 mg/kg bw [44]	0.000054	*Mixed*
Se	UL	0.255 mg [42]	0.0003175	*Saperavi*

PTWI = Provisional Tolerable Week Intake; TDI = Tolerable Daily Intake; BMDL = Benchmark Dose Lower Limit; UL = Upper Intake Level; PTMI = Provisional Tolerable Monthly Intake.

**Table 5:** Bioaccessibility of essential inorganic elements and phenolic compounds in grape pomace.

**Grape Pomace Varieties**		**Essential Inorganic Elements**						
		**Ca**	**Cu**	**Fe**	**Mg**	**Mn**	**P**	**Zn**
**Maximus**	Total content (mg/100 g)	251 ± 4	1.22 ± 0.04	6.87 ± 0.2	117 ± 1	2.68 ± 0.04	315 ± 3	0.63 ± 0.01
	Bioaccessible fraction (mg/100 g)	-	0.12 ± 0.02	-	33 ± 2	0.079 ± 0.01	81 ± 2	-
	Bioaccessibility (%)	-	10 ± 1 b	-	28 ± 2 a	2.90 ± 0.40 d	26 ± 1 a	-
**Saperavi DC**	Total content (mg/100 g)	336 ± 9	1.55 ± 0.03	5.54 ± 0.15	87 ± 1	6.16 ± 0.11	292 ± 4	1.57 ± 0.11
	Bioaccessible fraction (mg/100 g)	5.8 ± 0.6	0.086 ± 0.002	-	24 ± 1	0.86 ± 0.03	60 ± 4	-
	Bioaccessibility (%)	1.7 ± 0.2	5.5 ± 0.1 c	-	27 ± 1 a	13.9 ± 0.2 a	20 ± 1 b	-
**Pinot**	Total content (mg/100 g)	217 ± 1	8.31 ± 0.07	5.8 ± 0.3	112 ± 1	4.93 ± 0.08	301 ± 2	1.51 ± 0.07
	Bioaccessible fraction (mg/100 g)	-	1.1 ± 0.1	-	33 ± 1	0.49 ± 0.02	56 ± 4	-
	Bioaccessibility (%)	-	13 ± 1 a	-	30 ± 1 a	9.9 ± 0.4 c	18 ± 1 b	-
**Marselan**	Total content (mg/100 g)	207 ± 6	1.80 ± 0.01	5.98 ± 0.16	94 ± 1	5.18 ± 0.07	300 ± 5	1.75 ± 0.08
	Bioaccessible fraction (mg/100 g)	-	0.12 ± 0.01	-	28 ± 1	0.61 ± 0.03	61.1 ± 0.2	-
	Bioaccessibility (%)	-	6.8 ± 0.4 c	-	29 ± 1 a	12 ± 1 b	20.4 ± 0.1 b	-
**Merlot_2**	Total content (mg/100 g)	303 ± 5	9.62 ± 0.09	5.01 ± 0.22	106 ± 1	2.75 ± 0.03	271 ± 3	2.03 ± 0.06
	Bioaccessible fraction (mg/100 g)	-	1.24 ± 0.1	-	28 ± 1	0.029 ± 0.002	44 ± 9	-
	Bioaccessibility (%)	-	13 ± 1 a	-	27 ± 1 a	1.1 ± 0.1 e	16 ± 4 b	-
**Grape Pomace Varieties**	**Phenolic Compounds**							
	**Total Phenolics (mg GAE/100 g)**	**Phenolics GP** **(mg GAE/100 g)**		**GP Bioaccessibility (%)**		**Phenolics IP (mg GAE/100 g)**		**IP Bioaccessibility (%)**
**Maximus**	1595.3 ± 2.4	143.6 ± 0.3		9.0 bB		176.8 ± 0.3		11.1 bA
**Saperavi DC**	1842.8 ± 1.0	174.9 ± 0.1		9.5 bB		194.6 ± 0.4		10.6 bA
**Pinot**	1535.8 ± 3.4	211.7 ± 0.3		13.8 aB		294.5 ± 0.2		19.2 aA
**Marselan**	1298.2 ± 1.3	84.5 ± 0.1		6.5 cA		90.6 ± 0.2		6.9 cA
**Merlot_2**	1475.7 ± 0.8	87.8 ± 0.1		5.9 cB		155.9 ± 0.1		10.6 bA

Mean ± standard deviation (*n* = 3). GP: gastric phase; IP: intestinal phase. Results are expressed on a dry weight basis. Mean values with different lowercase letters in the same column indicate significant difference (*p* < 0.05) among samples, as determined by one-way ANOVA and Tukey test at 95% of confidence. Mean values with different capital letters in the same row indicate significant difference (*p* < 0.05) among stages of digestion, as determined by one-way ANOVA and Tukey test at 95% of confidence.

**Table 6:** Bioaccessibility of trace elements in grape pomace.

Grape Pomace Varieties		Al	Co	As	Ba
**Maximus**	Total content (µg/kg)	73,262 ± 3674	64 ± 7	43 ± 4	14,168 ± 114
	Bioaccessible fraction (µg/kg)	1671 ± 249	47 ± 7	38 ± 6	434 ± 50
	Bioaccessibility (%)	2.3 ± 0.3 c	72 ± 10 a	88 ± 14 a	3.1 ± 0.4 b
**Saperavi DC**	Total content (µg/kg)	76,601 ± 5926	48 ± 6	-	3824 ± 4
	Bioaccessible fraction (µg/kg)	3546 ± 47	17 ± 3	-	353 ± 44
	Bioaccessibility (%)	4.6 ± 0.1 b	36 ± 6 b	-	9 ± 1 a
**Pinot**	Total content (µg/kg)	54,269 ± 1180	30 ± 11	-	45,489 ± 1005
	Bioaccessible fraction (µg/kg)	2618 ± 310	12 ± 2	-	504 ± 67
	Bioaccessibility (%)	4.8 ± 0.6 b	42 ± 7 b	-	1.1 ± 0.1 c
**Marselan**	Total content (µg/kg)	92,845 ± 1707	53 ± 8	-	-
	Bioaccessible fraction (µg/kg)	3750 ± 169	15 ± 3	-	-
	Bioaccessibility (%)	4.0 ± 0.2 b	29 ± 6 b	-	-
**Merlot_2**	Total content (µg/kg)	23,150 ± 2152	-	43 ± 7	22,559 ± 493
	Bioaccessible fraction (µg/kg)	3068 ± 328	-	29 ± 7	502 ± 71
	Bioaccessibility (%)	13 ± 1 a	-	66 ± 17 a	2.2 ± 0.3 b,c

Mean ± standard deviation (*n* = 3). Means with different lowercase letters in the same column, indicate a significant difference (*p* < 0.05) determined using one-way ANOVA and Tukey test at 95% of confidence.

## Data Availability

The original contributions presented in this study are included in the article. Further inquiries can be directed to the corresponding author.

## Supplementary Materials

The following supporting information can be downloaded at: https://www.mdpi.com/article/10.3390/foods15122060/s1, Table S1: Operational parameters of the ICP OES and ICP-MS used for the determination of inorganic elements in grape pomace.
