# Supplementary material for: Potential Use of Grape Pomace for the Development of New Products: Study of Inorganic Elements Through Simulated Gastrointestinal Digestion
Source: Foods. 2026 Jun 7;15(12):2060. doi: 10.3390/foods15122060 (PMC13298386; doi:10.3390/foods15122060)
Supplement: Supplementary file 1 [file foods-15-02060-s001.zip › foods-4317872-supplementary.pdf]

Supplementary material:

Table S1. Operational parameters of the ICP OES and ICP-MS used for the determination of inorganic elements in grape pomace.

**ICP OES conditions**	
**RF Power**	1200 W
**Plasma gas flow / auxiliary gas flow**	12.0 / 1.0 L min^{-1}
**Spray chamber**	Cyclonic, double-pass
**Nebulizer**	Seaspray; 0.70 L min^{-1}
**Wavelength (nm)**	Ca (317.933), Cu (324.754), Fe (259.940), P (213.618), Mg (285.213), Mn (257.610), K (766.491), Na (589.592), Zn (206.200)
**ICP-MS conditions**	
**RF Power**	1550 W
**Plasma gas flow / auxiliary gas flow**	14.0 / 0.80 L min^{-1}
**Helium flow**	5.00 mL min^{-1}
**Nebulizer**	Micromist; 0.98 L min^{-1}
**Spray chamber**	Cyclonic, double-pass, at 2.8 °C
**Dwell time**	0.3 s / 0.02 s (PI)
**Monitored isotopes**	^{27}Al, ^{59}Co, ^{75}As, ^{78}Se, ^{97}Mo, ^{111}Cd, ^{123}Sb, ^{137}Ba, ^{202}Hg, ^{208}Pb
**Internal Standard (IS):** 50 μg L^{-1}	^{72}Ge; ^{103}Rh; ^{209}Bi; ^{195}Pt
